# Untargeted Metabolomics Analysis Revealed the Difference of Component and Geographical Indication Markers of *Panax notoginseng* in Different Production Areas

**DOI:** 10.3390/foods12122377

**Published:** 2023-06-15

**Authors:** Shijia Zhang, Kexin Fang, Zenan Ding, Jinxia Wu, Jianzhong Lin, Dunming Xu, Jinshui Zhong, Feng Xia, Jianghua Feng, Guiping Shen

**Affiliations:** 1 Department of Electronic Science, Fujian Provincial Key Laboratory of Plasma and Magnetic Resonance, Xiamen University, Xiamen 361005, China; 33320211150322@stu.xmu.edu.cn (S.Z.); 33320211150261@stu.xmu.edu.cn (K.F.); zenan_0519@163.com (Z.D.); 33320190153874@stu.xmu.edu.cn (J.W.); jszhong@xmu.edu.cn (J.Z.); xiafeng@xmu.edu.cn (F.X.); jianghua.feng@xmu.edu.cn (J.F.); 2 Technology Center of Xiamen Customs, Xiamen 361012, China; linjz@xmciq.gov.cn (J.L.); dunmingxu@163.com (D.X.)

**Keywords:** nuclear magnetic resonance, *Panax notoginseng*, quantitative component analysis, geographical indication marker, metabolomics

## Abstract

*Panax notoginseng* (*P. notoginseng*) has excellent medicinal and food dual-use characteristics. However, *P. notoginseng* with a unique origin label has become the target of fraud because of people confusing or hiding its origin. In this study, an untargeted nuclear magnetic resonance (NMR)-based metabolomics approach was used to discriminate the geographical origins of *P. notoginseng* from four major producing areas in China. Fifty-two components, including various saccharides, amino acids, saponins, organic acids, and alcohols, were identified and quantified through the NMR spectrum, and the area-specific geographical identification components were further screened. *P. notoginseng* from Yunnan had strong hypoglycemic and cardiovascular protective effects due to its high acetic acid, dopamine, and serine content, while *P. notoginseng* from Sichuan was more beneficial for diseases of the nervous system because of its high content of fumarate. *P. notoginseng* from Guizhou and Tibet had high contents of malic acid, notoginsenoside R1, and amino acids. Our results can help to distinguish the geographical origin of *P. notoginseng* and are readily available for nutritional recommendations in human consumption.

## 1. Introduction

*Panax notoginseng* (*P. notoginseng*) is a regional medicinal herb with powerful abilities to promote blood circulation and relieve pain [1]. Its distribution in China is mainly concentrated in the southwest, including Yunnan, Guangxi, and Sichuan provinces. *P. notoginseng* contains a wide range of effective nutritional components, such as *P. notoginseng* saponins, flavonoids, amino acids, volatile oils, plant box alcohols, sugars, inorganic salts, inorganic ions, and other active components, and thus exerts extensive pharmacological effects and clinical uses [2]. Saponins, the main active components of *P. notoginseng*, can stimulate the brain center, promote blood circulation, and enhance brain memory [3,4]. Flavonoids have been shown to reduce vascular fragility, improve the vascular permeability, lower blood lipids and cholesterol, and prevent and treat senile hypertension and cerebral hemorrhage [5]. *P. notoginseng* must be grown in warm, shady, and humid conditions to ensure high-quality production. Wenshan county in Yunnan Province of China is an important *P. notoginseng* producing area, where it enjoys a good reputation among consumers for its high quality. This reputation is closely related to the unique environmental conditions, such as the abundant rainfall and consistent annual temperature on the Yunnan–Guizhou plateau [6]. However, continuous crop obstacles also appear [1], leading to a sharp decline in or even total loss of yields. Therefore, the plant industry of *P. notoginseng* gradually expanded to the surrounding areas and other provinces and cities [7]. Although *P. notoginseng* plants from different producing areas have a similar appearance and fragrance, their characteristics and nutritional components vary to some extent due to regional factors such as climate, sun exposure, soil, precipitation, and humidity. Therefore, *P. notoginseng* with an origin label is more likely to become the target of potential fraud, which not only seriously disturbs the market order but also infringes on consumers’ rights [8]. Considering the growing illegal food trade, accurate traceability analysis of *P. notoginseng* is urgently needed, which is beneficial for the geographical origin and brand protection of high-quality products.

Some high-throughput analysis techniques have been applied to the origin identification and variety classification of Chinese herbal medicines, including liquid chromatography–mass spectrometry (LC-MS) [9], gas chromatography–mass spectrometry (GC-MS) [10], and DNA sequencing [11]. For example, Bai et al. established a characteristic fingerprint of *P. notoginseng* based on high performance liquid chromatography–mass spectrometry (HPLC-MS), and integrated the pharmacodynamic relationship to identify a variety of active ingredients in *P. notoginseng*, completing its origin identification [12]. However, a complicated pretreatment of samples, long running times, and poor reproducibility are usually present in these techniques. In recent years, nuclear magnetic resonance (NMR) technology combined with chemometrics has become the preferred technology for food analysis due to its advantages such as its non-destructive nature, quantitative results, and good reproducibility. In fact, NMR has been combined with chemometrics to perform the geographical identification and quality control of black pepper [13], China’s sweet orange [14], virgin olive oil [15], and quinoa [16]. In addition, it is also suitable for determining the metabolic effects of three plant growth regulators on strawberry ripening [17], and for identifying adulteration in Chinese monofloral honey [18] and Bordeaux red wines [19].

China is a major producer of *P. notoginseng*, and the illegal trade of *P. notoginseng*, such as its adulteration and unclear geographical origin, has recently increased around the nation [20]. Therefore, it is necessary to evaluate the geographical origin of *P. notoginseng*. In this study, high resolution NMR techniques were applied to determine the main components of *P. notoginseng* from the major producing areas in China, and the characteristic geographical indication markers of *P. notoginseng* from different producing areas were identified by multivariate statistical analysis.

## 2. Materials and Methods

### 2.1. Sample Collection and Preparation

*P. notoginseng* was provided by Technology Center of Xiamen Customs and collected in the local market from July to September 2020 in Southwest China, including Yanshan County, Wenshan City, Yunnan Province; Nanyang District, Guiyang City, Guizhou Province; Qingchuan County, Guangyuan City, Sichuan Province; and Naidong County, Shannan District, Tibet Autonomous Region (12 *P. notoginseng* samples per producing area, a total of 48 samples). All the *P. notoginseng* samples were ground into powder with a hammer mill (GH-20B, Jiangyin Kejia Machinery Manufacturing Co., Ltd., Jiangyin, China), dried in a ventilated oven at 70 °C for 24 h, and then sifted with a 60-mesh screen and stored in sealed plastic bags in the dark at 20 °C.

The powder samples of *P. notoginseng* were extracted by Bligh–Dyer method [21,22] to obtain the freeze-dried powder. In brief, *P. notoginseng* powder samples (300 mg) were homogenized at 4 °C for 30 s in 1.2 mL methanol and 0.6 mL deionized water. The homogenates were transferred to a 10 mL glass tube, and 1.2 mL of chloroform and 1.2 mL of deionized water were added into each tube, then vortexed for 60 s. After resting on ice for 15 min, the samples were centrifuged at 4 °C and 10,000× *g* for 10 min. All supernatants were transferred to 5.0 mL tubes, then nitrogen blown after freezing, and finally lyophilized for 24 h to remove the methanol, chloroform, and water. The extracts were stored at −80 °C for NMR experiments.

Before ^1^H-NMR analysis, freeze-dried powders of *P. notoginseng* were dissolved in 600 μL deuterated phosphate buffer (150 mM, pH 5.6) containing 0.05% sodium 3-(trimethylsilyl) propionate-2,2,3,3-d4 (TSP). The extracted *P. notoginseng* buffer mixture was kept at 25 °C for 5 min and then centrifuged for 10 min (10,000× *g*, 4 °C) to remove suspended debris. The supernatant (550 μL) was then transferred into a 5 mm NMR tube for NMR measurement.

### 2.2. ^1^H-NMR Spectra Acquisition and Processing

The NMR spectra of all *P. notoginseng* samples were performed on an 850 MHz Bruker Avance III NMR spectrometer (Bruker Corporation, Karlsruhe, Germany) equipped with a CPTCI probe operating at 850.13 MHz. The experiments were carried out by a pulse sequence of ZGPR at 298 K. The other parameters were set as: spectral width of 20 ppm, data points 16 K, 64 scans, 8 prior dummy scans, relaxation delay and acquisition time were 4 s and 1.58 s, respectively. In addition, three samples from each producing area were selected to collect a range of standard 2D NMR spectra to facilitate the subsequent accurate assignment of components in *P. notoginseng*, including COSY, TOCSY, HSQC, and HMBC.

The original data of *P. notoginseng* were preprocessed with MestReNova software (V9.0.1, Mestrelab Research, Santiago de Compostela, Galicia, Spain). Before Fourier transformation, all free induction decays were zero-filled to 64 K and processed by applying an exponential function with a line-broadening factor of 1.0 Hz, followed by phase and baseline correction, and the entire spectrum was corrected with TSP at δ0.0. The spectral interval of δ0.6–9.6 was sectionally integrated with an interval of 0.002 ppm. The baseline of all the spectrum peak ranges and the residual water resonance (δ4.66–4.92) were removed to eliminate baseline effects of water signals. The spectral interval of δ0.6–9.6 was sectionally integrated with an integral width of 0.002 ppm, then normalized to a total area of 100.

### 2.3. Statistical Analysis

The raw integral data were imported into SIMCA 14.1 (Umetrics, Umea, Sweden) software for multivariate statistical analysis, including unsupervised principal component analysis (PCA) for explaining natural distribution trends, partial least squares discriminant analysis (PLS-DA), and supervised orthogonal partial least squares discriminant analysis (OPLS-DA) for the identification of geographical indication components in *P. notoginseng* corresponding to the different producing areas. Parameters R^2^ and Q^2^ were used to evaluate the fitting performance and predictive ability of the model, respectively [23]. A 900-times permutation test was then conducted to validate whether the models were overfitted. Moreover, cross-validation analysis of the variance (CV-ANOVA) was performed to access significance of the models. *p* < 0.05 was considered to be statistically significant in all experiments.

Finally, a four-dimensional volcano plot was generated with MATLAB scripts (downloaded from http://www.mathworks.com (accessed on 27 June 2020)) with some in-house modifications to screen biomarkers using the geographical indication components of *P. notoginseng* from different geographical origins with a combination of univariate and multivariate statistical analyses. The univariate statistical analyses were performed by using the fold-change value and Student’s *t*-test, where the fold-change was equal to the concentration ratio of each component in *P. notoginseng* between one special producing area and the three other areas, and the *t*-test was converted to *p*-values to assess and confirm the significant change in each component. In this study, a four-dimensional volcano plot, which is a scatter plot of −log10 (*p*-value) against log2 (fold-change), was applied to identify the distinguished metabolites between pairwise groups. The absolute correlation coefficient values r and the VIP values from the multivariate analysis served as two important indicators represented by circle color and circle size in volcano plot, respectively (warmer color symbolizes a higher |r|, and a larger circle size symbolizes a higher VIP value). The components that exhibited significant changes were screened out by combing the restrictions of three criteria: *p* < 0.05, |r| > 0.5, and VIP values in the top 10%, which was segmented by the horizontal threshold line *p* = 0.05 and tended to be located in upper zones of the plots with the larger circle sizes and warmer colors. On this basis, the components with a 1.2-fold increase in content and a correlation coefficient |r| greater than 0.65 were defined as geographical indication markers.

In addition, the absolute concentration of each component in *P. notoginseng* was accurately quantified by comparing integral of the characteristic peak with that of the internal standard (TSP) [14].

$$C_{x}=\frac{A_{x}}{N_{x}}\times \frac{N_{\mathrm{TSP}}}{A_{\mathrm{TSP}}}\times C_{\mathrm{TSP}}$$

where C_x_ is the molar concentration of any component x in *P. notoginseng*, in mol/L; A_x_ is the integral area of the characteristic peak of component x in the ^1^H-NMR spectrum; N_x_ is the number of hydrogens contributing to the NMR signals; N_TSP_ is the number of hydrogens corresponding to the singlet at δ0.00 (here, N_TSP_ = 9); A_TSP_ is the integral area of TSP at δ0.00; and C_TSP_ is the molar concentration of TSP in *P. notoginseng*, in mol/L.

## 3. Results and Discussion

### 3.1. ^1^H-NMR Analysis of P. notoginseng from Different Areas

**Figure 1:**
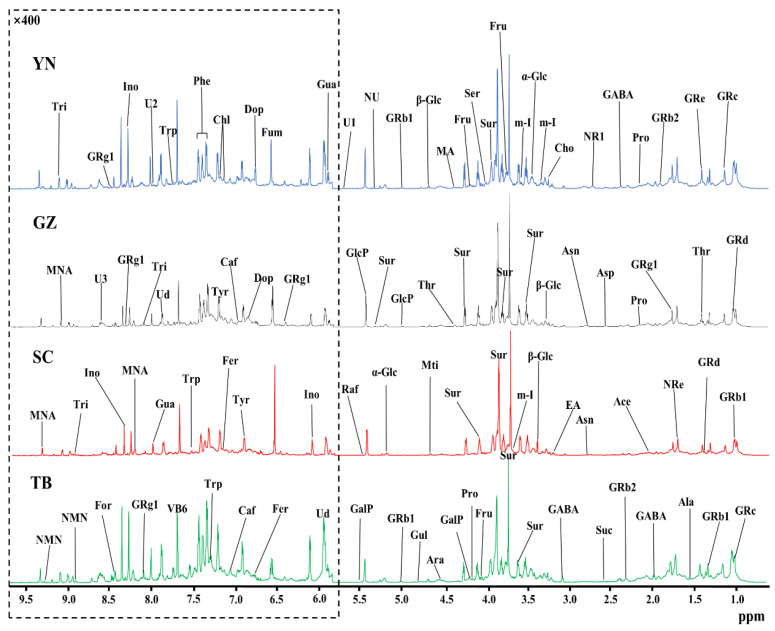
An 850 MHz ^1^H-NMR spectral comparison of *P. notoginseng* from four producing areas: Yunnan (YN); Guizhou (GZ); Sichuan (SC); and Tibet (TB). For clear observation, the spectral regions of δ6.0–9.5 were vertically magnified 400 times relative to the spectral regions of δ0.5–5.5. The keys and the details of the labeled spectral information of the components and the corresponding quantitative results in each producing area are summarized in Table 1.

The high resolution one-dimensional ^1^H-NMR spectrum provided a clear metabolic overview of *P. notoginseng* from different producing areas. Therefore, the differences in the components of *P. notoginseng* from different producing areas were visualized by the spectral peaks of different chemical components in the normalized ^1^H-NMR spectrum. The stacked NMR spectra of *P. notoginseng* from four different areas in Yunnan (YN), Guizhou (GZ), Sichuan (SC), and Tibet (TB) are compared in Figure 1. The spectra were assigned according to chemical shift, peak multiplicity, and related literature data [24], and further confirmed by 2D NMR (COSY, TOCSY, HSQC, and HMBC NMR spectra), the public Biologic Magnetic Resonance Database (http://bmrb.protein.osaka-u.ac.jp/deposit (accessed on 10 June 2020), BMRB), and the Traditional Chinese Medicine Systematic Pharmacology Database (http://tcmspw.com/tcmsp.php (accessed on 10 June 2020), TCMSP) [25,26,27]. Fifty-two components were assigned from the ^1^H-NMR spectra, and three signals were not identified. Table 1 lists the spectral information in detail, including chemical shift and peak multiplicity. By comparing the integral of the characteristic peak and internal standard (TSP), the concentrations of each component in *P. notoginseng* from different producing areas were quantitatively obtained. Low signal to noise (S/N) ratios and obvious peak overlap could hinder the correct quantification of the components in *P. notoginseng*. Therefore, in order to achieve accurate quantification, only the resonances that were well resolved or at least dominant in local regions of each component were chosen as characteristic resonances (underlined peaks in Table 1) for accurate quantification. In addition, an S/N = 10 was set as the quantitative critical threshold. Finally, 52 components across the four geographical origins of *P. notoginseng* were quantified, and their signal information was selected for quantification. The quantitative results are tabulated in Table 1.

As shown in Figure 1, *P. notoginseng* from different producing areas are rich in various *P. notoginseng* saponins and ginsenosides that are distributed throughout the spectrum (δ1.0–9.5). In addition, the signal of these saponins was strong, confirming that *P. notoginseng* from the four producing areas was in accordance with the basic nutritional characteristics of *P. notoginseng*. Further analysis of the spectra showed that a variety of amino acids, such as threonine (Thr, δ1.32), alanine (Ala, δ1.47), γ-aminobutyric acid (GABA, δ1.91), and proline (Pro, δ1.99) were found in the aliphatic region (δ0.5–3.0) but at relatively low levels. The components with stronger intensities were found in the carbohydrate region (δ3.0–6.0), indicating that the carbohydrate content was higher in *P. notoginseng*. Among them, sucrose (Sur, δ3.67 and 3.87) showed the highest content. The relative contents of α-glucose (α-Glc), β-glucose (β-Glc), and fructose (Fru) were also high, and contributed to the sweet taste of *P. notoginseng*. However, the signal of the aromatic region (δ6.0–9.5) was much weaker than that of the aliphatic region. In order to clearly show the component peaks of the aromatic region, the δ6.0–9.5 region in Figure 1 was vertically magnified 400 times. The visibly stronger signals were uridine (Ud, δ5.89, 5.91, and 7.87), fumarate (Fum, δ6.53), pyridoxine (vitamin B6, δ7.67), inosine (Ino, δ6.06 and 8.35), and 1-methylnicotinamide (MNA, δ8.21 and 9.34).

The *P. notoginseng* from the different producing areas displayed similar metabolic profiles, implying that the nutritional compositions were similar. The main components were carbohydrates and saponins, but it was difficult to distinguish *P. notoginseng* from the four main producing areas only by comparing the NMR peaks of the carbohydrate compounds and saponins due to their approximate ratios. In fact, further analysis showed that the signals of low-content components in the *P. notoginseng* from different producing areas varied considerably, and thus made greater contributions to geographical indication. For example, the contents of fumarate (δ6.53) in *P. notoginseng* from SC and uridine (δ5.89) from TB were remarkably higher than from the other three producing areas, while the pyridoxine content (vitamin B6, δ7.67) of GZ *P. notoginseng* was clearly lower than in the other producing areas. It is difficult to distinguish the origin of *P. notoginseng* solely by relying on a visual comparison of the content of the components in the spectrum. Thus, pattern recognition methods were used to enable us to identify the geographical indicators of *P. notoginseng* in the four producing areas.

### 3.2. Geographical Origin Discrimination of P. notoginseng

**Figure 2:**
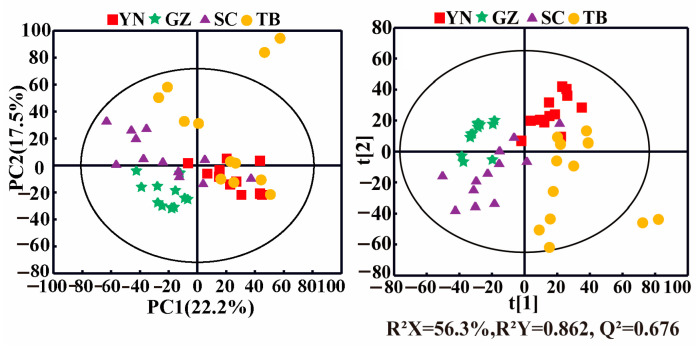
PCA (**left**) and PLS-DA (**right**) score plots of *P. notoginseng* from different producing areas. (YN: Yunnan; GZ: Guizhou; SC: Sichuan; TB: Tibet).

PCA can identify possible outliers in samples, and it helped visualize the differences and similarities of *P. notoginseng* from the different producing areas in this study. A PCA score plot (Figure 2, left panel) showed the clustering of samples from the same geographical origin, although overlaps were present between different producing areas. For example, the *P. notoginseng* sample points from YN, SC, and TB were adjacent and overlapped with each other but were distinguished from the samples of GZ. The corresponding PLS-DA score plot (Figure 2, right panel) showed the separations of the *P. notoginseng* samples from the different producing areas with the good fitting performance (R^2^ Y = 0.862) and favorable predictive ability (Q^2^ = 0.676). In general, the samples of *P. notoginseng* from the four producing areas were roughly distributed in four areas of the PLS model. However, a slight overlap existed between the samples from SC, YN, and TB, suggesting their similar but distinguishing nutrient compositions. YN, SC, and TB are geographically adjacent to each other, resulting in similar compositional characteristics of *P. notoginseng*, while the differences in climate, altitude, and rainfall led to distinct differences in *P. notoginseng* nutrients. In addition, compared with the other producing areas, the samples of *P. notoginseng* in the TB and SC groups were dispersed, indicating that the composition of *P. notoginseng* in TB and SC was greatly affected by external factors.

### 3.3. Identification of Geographical Indication Components of P. notoginseng

**Figure 3:**
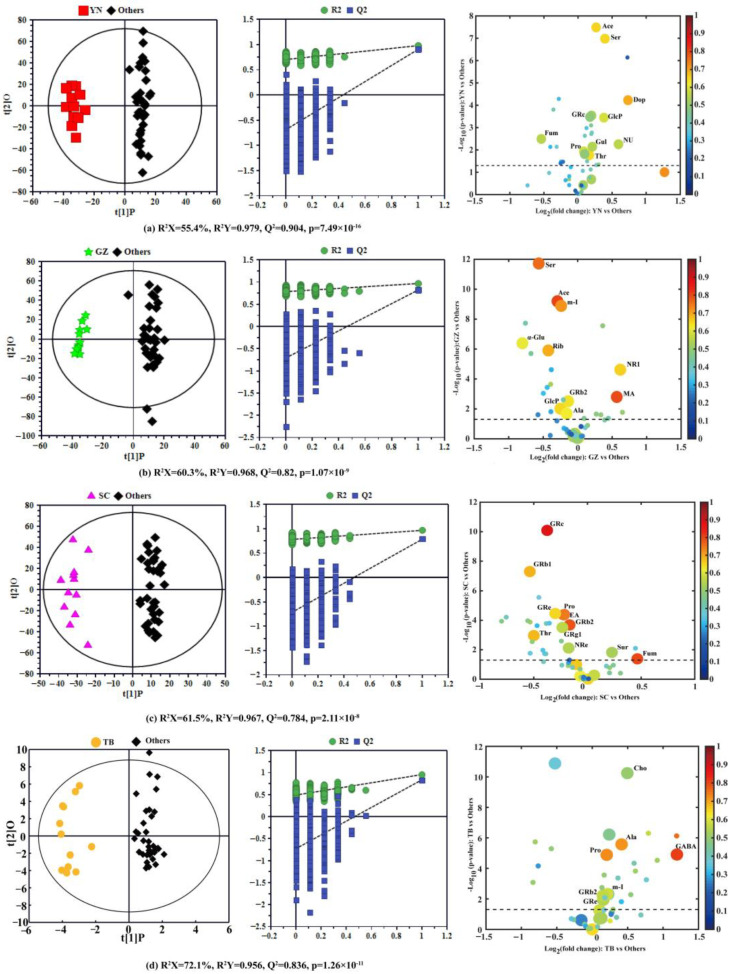
OPLS-DA score plots (left), 900-times permutation test plots (middle), and the corresponding volcano plots (right) derived from the NMR data of *P. notoginseng* from the different producing areas. (**a**) Yunnan vs. the three other producing areas; (**b**) Guizhou vs. the three other producing areas; (**c**) Sichuan vs. the three other producing areas; (**d**) Tibet vs. the remaining three producing areas. (YN: Yunnan; GZ: Guizhou; SC: Sichuan; TB: Tibet; Others: the other three producing areas).

To further explore the compositional differences of *P. notoginseng* and find significant geographical markers of *P. notoginseng* from the different producing areas, four pairwise comparison OPLS-DA models were constructed in the same variety (12 *P. notoginseng* samples per producing area, a total of 48 samples) between each special geographical origin and the other three origins. The OPLS-DA score plot with a confidence level of 95% (left panels), 7-fold cross validation and permutation tests (permutation number *n* = 900) (middle panels), and the corresponding volcano plots (right panels) derived from the NMR data of *P. notoginseng* are shown in Figure 3, and the model parameters, including R^2^ X, R^2^ Y, and Q^2^, are also provided. All models use the first prediction and four orthogonal (1 + 4) components and were also further validated by CV-ANOVA (Figure 3).

The OPLS-DA model for YN compared with the other three producing areas is shown in Figure 3a (left), with two sets of sample points clearly separated on both sides of the longitudinal axis. The high predictive ability (Q^2^ = 0.979), good fitting performance (R^2^ Y = 0.904), and very low *p*-value from CV-ANOVA (7.49 × 10^−16^) further revealed the remarkable difference in nutrient composition of *P. notoginseng* between YN and the other three regions. The geographical indications corresponding to the specific producing area were identified according to the screening criteria identified in the Materials and Methods section. The volcano plot in Figure 3a (right) displays the ten potential geographical indication components of *P. notoginseng* in YN, and the detailed statistical parameters of these components are summarized in Table 2. Ten components in YN *P. notoginseng*, including acetic acid, dopamine, ginsenoside Rc, pentasonoside U, glucosamine 6-phosphate, fumarate, proline, corollactone, serine, and threonine, were significantly different from those in the other three producing areas. Among them, acetic acid (|r| = 0.66), dopamine (|r| = 0.70), serine (|r| = 0.65), and threonine (|r| = 0.65) were potential geographical markers of YN *P. notoginseng*. Furthermore, the contents of acetic acid (FC = 1.20), dopamine (FC = 1.66), glucosamine 6-phosphate (FC = 1.30), *P. notoginseng* U (FC = 1.51), and serine (FC = 1.31) were significantly higher in *P. notoginseng* from YN than from the other producing areas. However, the relative content of fumarate in YN *P. notoginseng* (FC = 0.69) was much lower than in *P. notoginseng* from other areas. Studies have demonstrated that acetic acid has a certain antagonistic influence on type II diabetes caused by obesity [28]; serine plays a dominant role in ensuring the normal development of the central nervous system [29]; and dopamine is considered to be an essential regulator of central and peripheral biological functions in humans and animals [30] and plays an important role in cardiovascular regulation [31]. Considering the definition of a geographical indication marker, acetic acid (|r| = 0.66, FC = 1.20), dopamine (|r| = 0.70, FC = 1.66), and serine (|r| = 0.65, FC = 1.31) were chosen as geographical indication markers of YN *P. notoginseng*.

The OPLS-DA model (Figure 3b (left)) of GZ vs. the other three geographical origins produced a more distinct separation according to the model parameters (R^2^ Y = 0.968 and Q^2^ = 0.820); the permutation test and the quite low *p*-value (1.07 × 10^−9^) from CV-ANOVA also further indicate their differences in composition. The corresponding volcano plot (Figure 3b (right)) and the parameters of various components (Table 2) indicated that threonine, alanine, malic acid, acetic acid, inositol, α-glucose, ribolactone, raffinose, notoginsenoside R1, ginsenoside Rb2, and glucosamine 6-phosphate in GZ *P. notoginseng* were significantly different from *P. notoginseng* of the other three producing areas. Except for malic acid and *P. notoginseng* saponins, all the other potential components had positive correlation coefficients (r > 0). In addition, except for alanine (|r| = 0.63) and ginsenoside Rb2 (|r| = 0.64), the correlation coefficients of the other eight potential components were greater than 0.65 (|r| ≥ 0.65). The contents of malic acid and notoginsenoside R1 in GZ *P. notoginseng* were higher than those of *P. notoginseng* from the other regions (FC > 1), while the contents of the other eight components were lower. One study has shown that notoginsenoside R1 has multiple effects, such as hemostatic coagulation, hypolipidemic, antithrombotic, immune function, and cardiovascular protection [32]. In fact, malic acid has been favored by the cosmetics industry, mainly to adjust the pH of cosmetics, skin conditioning, and moisturizers, and has been used in nearly 50 cosmetic formulations [33]. According to the selected criteria mentioned above, our study showed that the higher content components of malic acid (|r| = 0.80, FC = 1.48) and *P. notoginseng* saponin R1 (|r| = 0.66, FC = 1.53) could be selected as GZ *P. notoginseng* geographical indication markers.

Figure 3c reveals the comparison between SC *P. notoginseng* and those in other special geographical origins. The OPLS-DA model (Figure 3c (left)) displayed the clear separation between the two sets of samples, and the model produced high statistical values of R^2^ Y (0.967) and Q^2^ (0.784), and the quite low *p*-value from CV-ANOVA (2.11 ×10^−8^) proved the obvious difference in the nutritional ingredients of *P. notoginseng* from SC compared to the other producing areas. The corresponding volcano plot (Figure 3c (right)) and parameters of various components (Table 2) revealed that there were eleven significant differential components between *P. notoginseng* from SC and the other regions, including ginsenosides Rb1, Rb2, Rc, Re, Rg1, ginsenoside Re, proline, threonine, fumarate, sucrose, and ethanolamine. It can be seen from Table 2 that only fumarate and sucrose had negative correlation coefficients, and ginsenoside Rg1 (|r| = 0.56), ginsenoside Re (|r| = 0.55), and sucrose (|r| = 0.54) had weak correlations (|r| < 0.65). Furthermore, the contents of fumarate and sucrose in *P. notoginseng* from SC province were higher than in *P. notoginseng* from the other producing areas (FC > 1), and were located in the positive region of the x-axis, as shown in Figure 3c (left). Researches have shown that fumarate plays a vital role in coping with diseases of the nervous system by modulating neurons [34]. Similarly, through this comprehensive analysis, fumarate (|r| = 0.80, FC = 1.38) can be used as a geographical marker of *P. notoginseng* in SC.

The OPLS-DA score plot displayed an obvious and substantial separation of *P. notoginseng* samples between TB and the other three producing areas (Figure 3d (left)) with high statistical values of R^2^ Y (0.956) and Q^2^ (0.836), which was further supported by the permutation test and the quite low *p*-value from CV-ANOVA (1.26 × 10^−11^). According to the volcano plot (Figure 3d (right)), there were significant differences in seven components, including choline, alanine, proline, γ-aminobutyric acid, inositol, and ginsenoside Rb2 and Re between *P. notoginseng* from Tibet and the other regions, and these seven components had negative correlations (r < 0) (Table 2), while alanine (|r| = 0.72), γ-aminobutyric acid (|r| = 0.82), and proline (|r| = 0.73) had positive correlations (|r| > 0.65). Further analysis of the relative content change (FC value) of these seven components showed a higher abundance in TB *P. notoginseng* than in the others (FC > 1). Among them, the relative content of γ-aminobutyric acid was the highest, and was 2.29 times that of the other *P. notoginseng*, and choline and alanine were 1.41 and 1.33 times that of the other *P. notoginseng*, respectively. These components with significant differences were all located in the positive region of the x-axis (right panel of Figure 3d). Studies have found that alanine has a potentially supportive effect for maintaining basic brain function in hypoglycemia, and γ-aminobutyric acid, one of the most famous neurotransmitter molecules [35], has a positive effect on inhibiting neural excitability. It has multiple physiological functions, such as promoting brain activity, relieving pain, and improving sleep [36,37]. Similarly, alanine (|r| = 0.72, FC = 1.33) and γ-aminobutyric acid (|r| = 0.82, FC = 2.29), with a high content and high correlation coefficients, could be considered as geographical indication markers of TB *P. notoginseng*.

The geographical indication components of *P. notoginseng* corresponding to specific geographical origins are generalized in Table 2. It can be observed that *P. notoginseng* from different producing origins displayed unique nutritional characteristics. For example, YN *P. notoginseng* has an excellent cardiovascular function due to its rich dopamine compounds, and SC *P. notoginseng* has a positive effect on neuron regulation due to its high content of fumarate. However, the potential impact of batch, season, and production year on the components of *P. notoginseng* was not considered in this study. In future work, these effects ought to be further investigated to validate and modify the conclusions.

## 4. Conclusions

In this study, an inexpensive and convenient untargeted metabolomics approach based on NMR technology was used to conduct origin traceability on *P. notoginseng* from four geographical origins in China. Fifty-two main components including amino acids, saponins, sugars, alcohols, and organic acids were analyzed and quantified from the ^1^H-NMR spectrum of *P. notoginseng*. A combination of many multivariate statistical analyses, including PCA, PLS-DA, OPLS-DA, and ^1^H-NMR, was successfully applied to the classification and visualization of *P. notoginseng* in Yunnan, Guizhou, Sichuan, and Tibet, and to further identify the geographical indication components of Chinese *P. notoginseng* corresponding to their specific geographical origins. The quantification of a wealth of various nutrients in *P. notoginseng* can be used for nutrition recommendations for human consumption. The geographical indication markers can help to quickly classify the geographical sources of *P. notoginseng* according to their nutritional characteristics. The results of this experiment indicated that NMR combined with pattern recognition could be used as an effective method to trace the geographical origin of *P. notoginseng* and provide a reference for the analysis and identification of other Chinese herbs.

## Figures and Tables

**Table 1:** The components identified by NMR spectroscopy and quantitative results of *P. notoginseng* from different producing areas.

Components	Abbr.	Chemical Shift (ppm) (Multiplicity)	Concentration (mM)			
			YN ^a^	GZ	SC	TB
1-Methylnicotinamide	MNA	8.21 (t ^b^), 9.34 (s) ^c^	/ ^d^	/	/	/
Acetic acid	Ace	1.94 (s)	5.25 ± 0.02 ^e^	3.96 ± 0.02	4.37 ± 0.05	4.97 ± 0.02
Alanine	Ala	1.47 (d)	4.54 ± 0.03	4.08 ± 0.04	3.90 ± 0.05	5.56 ± 0.04
Arabinose	Ara	4.48 (d)	6.44 ± 0.03	5.78 ± 0.04	5.35 ± 0.10	5.85 ± 0.13
Asparagine	Asn	2.85 (dd), 2.95 (dd)	0.58 ± 0.02	0.57 ± 0.01	0.62 ± 0.01	0.74 ± 0.02
Aspartic acid	Asp	2.58 (dd), 2.68 (dd)	1.07 ± 0.01	0.89 ± 0.02	1.19 ± 0.02	1.30 ± 0.02
Caffeic acid	Caf	6.92 (d), 7.04 (m)	0.089 ± 0.001	0.103 ± 0.003	0.068 ± 0.002	0.106 ± 0.002
Chlorogenic acid	Chl	6.95 (m), 7.11 (m)	0.075 ± 0.001	0.104 ± 0.003	0.069 ± 0.003	0.100 ± 0.002
Choline	Cho	3.19 (s)	6.45 ± 0.02	5.24 ± 0.02	4.60 ± 0.07	7.65 ± 0.03
Dopamine	Dop	6.74 (dd), 6.84 (d)	0.156 ± 0.003	0.104 ± 0.002	0.084 ± 0.004	0.093 ± 0.003
Ethanolamine	EA	3.15 (t)	6.11 ± 0.02	5.75 ± 0.03	5.19 ± 0.04	6.38 ± 0.03
Ferulic acid	Fer	6.82 (d), 7.15 (d)	0.077 ± 0.001	0.101 ± 0.002	0.070 ± 0.002	0.100 ± 0.002
Formic acid	For	8.44 (s)	0.034 ± 0.001	0.031 ± 0.001	0.049 ± 0.003	0.061 ± 0.003
Fructose	Fru	3.99 (m), 4.12 (d), 3.70 (m)	3.21 ± 0.03	3.17 ± 0.11	3.39 ± 0.08	4.19 ± 0.04
Fumarate	Fum	6.53 (s)	0.155 ± 0.004	0.256 ± 0.008	0.258 ± 0.008	0.132 ± 0.003
Galactose 1 phosphate	GalP	4.18(t), 5.50 (d)	0.234 ± 0.005	0.206 ± 0.005	0.184 ± 0.003	0.173 ± 0.006
γ-Aminobutyric acid	GABA	1.91 (q), 2.32 (t), 3.01 (t)	2.46 ± 0.02	2.48 ± 0.04	2.45 ± 0.05	5.64 ± 0.07
Ginsenoside Rb1	GRb1	0.95 (s), 1.25 (s), 5.02 (d), 5.16 (br)	19.67 ± 0.11	18.87 ± 0.22	12.45 ± 0.18	20.25 ± 0.16
Ginsenoside Rb2	GRb2	1.84 (s), 2.24 (s)	12.91 ± 0.08	11.03 ± 0.07	10.87 ± 0.07	12.88 ± 0.08
Ginsenoside Rc	GRc	0.93 (s), 1.07 (br)	30.77 ± 0.20	27.31 ± 0.21	22.93 ± 0.15	31.89 ± 0.11
Ginsenoside Rd	GRd	0.97 (s), 1.28 (s)	20.11 ±0.14	20.60 ± 0.16	21.90 ± 0.41	22.81 ± 0.29
Ginsenoside Re	GRe	1.34 (s)	17.24 ± 0.07	17.44 ± 0.10	14.25 ± 0.15	18.17 ± 0.13
Ginsenoside Rg1	GRg1	1.70 (s), 6.37 (br), 8.09 (s), 8.30 (s) 8.47 (s)	23.48 ± 0.22	22.09 ± 0.21	19.48 ± 0.19	23.27 ± 0.24
Glucosamine 6-phosphate	GlcP	4.96 (d), 5.45 (d)	1.57 ± 0.02	1.15 ± 0.01	1.07 ± 0.01	1.50 ± 0.05
Guanosine	Gua	5.85 (d), 7.99 (s)	0.074 ± 0.001	0.055 ± 0.001	0.056 ± 0.001	0.093 ± 0.001
Gulonolactone	Gul	4.73 (d)	1.84 ± 0.02	1.63 ± 0.02	1.53 ± 0.02	1.64 ± 0.03
Inosine	Ino	6.06 (d), 8.35 (s)	0.122 ± 0.002	0.074 ± 0.001	0.080 ± 0.001	0.174 ± 0.002
Malic acid	MA	4.33 (dd)	2.51 ± 0.03	3.73 ± 0.09	2.86 ± 0.06	2.03 ± 0.03
Maltotriose	Mti	4.65 (d)	0.85 ± 0.01	0.405 ± 0.004	0.59 ± 0.02	0.57 ± 0.01
Myo-Inositol	m-I	3.27 (t), 3. 52 (dd), 3.61 (t)	11.10 ± 0.03	9.23 ± 0.02	10.09 ± 0.09	11.78 ± 0.10
N-Methylnicotinamide	NMN	8.90 (d), 9.29 (s)	/	/	/	/
Notoginsenoside R1	NR1	2.74 (d)	3.20 ± 0.07	3.89 ± 0.06	2.36 ± 0.07	1.81 ± 0.04
Notoginsenoside Re	NRe	1.64 (br)	41.59 ± 0.21	39.48 ± 0.33	36.22 ± 0.41	41.99 ± 0.28
Notoginsenoside U	NU	5.29 (s)	1.31 ± 0.04	0.96 ± 0.02	0.95 ± 0.01	0.60 ± 0.02
Phenylalanine	Phe	7.32 (d), 7.37 (m), 7.42 (m)	0.201 ± 0.001	0.245 ± 0.006	0.180 ± 0.005	0.361 ± 0.003
Proline	Pro	1.99 (m), 2.06 (m), 4.13 (m)	14.78 ± 0.04	13.94 ± 0.07	12.64 ± 0.10	15.88 ± 0.06
Pyridoxine (Vitamin B6)	VB6	7.67 (s)	0.173 ± 0.002	0.154 ± 0.003	0.189 ± 0.007	0.166 ± 0.008
Raffinose	Raf	5.43 (d)	0.83 ± 0.01	0.80 ± 0.02	0.93 ± 0.02	0.77 ± 0.01
Ribonolactone	Rib	4.42 (dd)	2.03 ± 0.02	1.57 ± 0.01	1.82 ± 0.03	2.63 ± 0.05
Serine	Ser	3.95 (dd)	6.90 ± 0.05	4.23 ± 0.03	5.60 ± 0.09	6.26 ± 0.06
Succinic acid	Suc	2.51 (s)	2.16 ± 0.04	2.86 ± 0.11	1.37 ± 0.05	2.00 ± 0.05
Sucrose	Sur	3.46 (t), 3.55(dd), 3.67 (s), 3.75 (s), 3.82 (m), 3.87 (dd), 4.04 (t), 4.21 (d), 5.40 (d)	19.04 ± 0.08	26.29 ± 0.12	24.66 ± 0.33	16.21 ± 0.09
Threonine	Thr	1.32 (d), 4.27 (m)	7.61 ± 0.04	7.53 ± 0.10	5.38 ± 0.15	7.74 ± 0.04
Trigonelline	Tri	8.12 (m), 8.94 (t), 9.19 (m)	/	/	/	/
Tryptophan	Trp	7.27 (m), 7.52 (d), 7.72 (d)	0.042 ± 0.001	0.039 ± 0.001	0.030 ± 0.001	0.085 ± 0.001
Tyrosine	Tyr	6.89 (d), 7.19 (d)	0.197 ± 0.001	0.244 ± 0.004	0.210 ± 0.006	0.331 ± 0.004
Unknown1	U1	5.79 (d)	/	/	/	/
Unknown2	U2	7.89 (d)	/	/	/	/
Unknown3	U3	8.61 (s)	/	/	/	/
Uridine	Ud	5.89 (d), 5.91 (d), 7.87 (d)	0.129 ± 0.002	0.094 ± 0.03	0.093 ± 0.04	0.178 ± 0.03
α-Glucose	α-Glc	3.42 (t), 5.22 (d)	1.52 ± 0.02	0.98 ± 0.03	1.88 ± 0.04	1.75 ± 0.03
β-Glucose	β-Glc	3.23 (dd), 3.41 (t), 4.63 (d)	2.28 ± 0.03	1.88 ± 0.05	2.75 ± 0.07	3.06 ± 0.06

^a^ YN: Yunnan, GZ: Guizhou, SC: Sichuan, TB: Tibet. ^b^ Multiplicity: s, singlet; d, doublet; t, triplet; q, quartet; dd, doublet. m, multiplet; br, broad spectrum; ^c^ Underlined peaks indicate the characteristic signals of each component in the *P. notoginseng* for the quantitative analysis. ^d^ Not available for the quantitative analysis. ^e^ Quantitative results are presented as mean ± standard deviation and calculated from 12 samples in the same group.

**Table 2:** Summary of statistical parameters of the differential components in *P. notoginseng* from different producing areas.

Components	YN-Others				GZ-Others				SC-Others				TB-Others			
	r ^a^	FC ^b^	*p* ^c^	VIP ^d^	r	FC	*p*	VIP	r	FC	*p*	VIP	r	FC	*p*	VIP
Acetic acid	−0.66	1.20	3.3 × 10^−8^	1.80	0.81	0.82	6.4 × 10^−10^	0.96	/ ^e^	/	/	/	/	/	/	/
Alanine	/	/	/	/	0.63	0.90	0.02	1.23	/	/	/	/	−0.72	1.33	2.7 × 10^−6^	1.56
Choline	/	/	/	/	/	/	/	/	/	/	/	/	−0.51	1.41	5.7 × 10^−11^	2.00
Dopamine	−0.70	1.66	5.9 × 10^−5^	1.90	/	/	/	/	/	/	/		/	/	/	/
Ethanolamine	/	/	/	/	/	/	/	/	0.83	0.86	4.1 × 10^−5^	2.21	/	/	/	/
Fumarate	−0.55	0.69	3.2 × 10^−3^	1.49	/	/	/	/	−0.80	1.38	0.04	2.18	/	/	/	/
γ-Aminobutyric acid	/	/	/	/	/	/	/	/	/	/	/	/	−0.82	2.29	1.2 × 10^−5^	2.49
Ginsenoside Rb1	/	/	/	/	/	/	/	/	0.69	0.69	5.1 × 10^−8^	1.94	/	/	/	/
Ginsenoside Rb2		/	/	/	0.64	0.91	0.003	1.29	0.79	0.89	2.1 × 10^−4^	2.16	−0.57	1.11	0.006	1.28
Ginsenoside Rc	−0.53	1.15	2.9 × 10^−4^	1.53	/	/	/	/	0.89	0.77	8.2 × 10^−11^	2.40	/	/	6.11 × 10^−7^	2.70
Ginsenoside Re	/	/	/	/	/	/	/	/	0.65	0.81	3. 5 × 10^−5^	1.82	−0.55	1.11	0.01	3.09
Ginsenoside Rg1	/	/	/	/	/	/	/	/	0.56	0.85	3.1 × 10^−4^	1.63	/	/	/	/
Glucosamine-6-phosphate	−0.59	1.30	3.6 × 10^−4^	1.68	0.65	0.84	0.01	1.39	/	/	/	/	/	/	/	/
Gulonolactone	−0.55	1.15	7.2 × 10^−3^	1.54	/	/	/	/	/	/	/	/	/	/	/	/
Malic acid	/	/	/	/	−0.80	1.48	0.002	2.04	/	/	/	/	/	/	/	/
Myo-Inositol	/	/	/	/	0.72	0.85	1.3 × 10^−9^	1.53	/	/	/	/	−0.59	1.16	0.005	1.63
Notoginsenoside R1	/	/	/	/	−0.66	1.53	2.4 × 10^−5^	1.28	/	/	/	/	/	/	/	/
Notoginsenoside Re	/	/	/	/	/	/	/	/	0.55	0.89	0.008	1.61	/	/	/	/
Notoginsenoside U	−0.56	1.51	5.6 × 10^−3^	1.55	/	/	/	/	/	/	/	/	/	/	/	/
Proline	−0.59	1.06	0.01	1.67	/	/	/	/	0.75	0.86	4.3 × 10^−5^	2.06	−0.73	1.15	1.3 × 10^−5^	1.55
Raffinose	/	/	/	/	/	/	/	/	/	/	/	/	/	/	/	/
Ribonolactone	/	/	/	/	0.70	0.74	1.2 × 10^−6^	1.50	/	/	/	/	/	/	/	/
Serine	−0.65	1.31	1.0 × 10^−7^	1.79	0.77	0.68	1.9 × 10^−12^	1.69	/	/	/	/	/	/	/	/
Sucrose	/	/	/	/	/	/	/	/	−0.54	1.17	0.02	1.51	/	/	/	/
Threonine	−0.65	1.12	0.02	1.79	/	/	/	/	0.68	0.71	0.001	1.92	/	/	/	/
α-Glucose	/	/	/	/	0.61	0.57	4.1 × 10^−7^	0.99	/	/	/	/	/	/	/	/

^a^ r value represents the correlation coefficient, |r| > 0.5 represents significant correlation. ^b^ FC, fold-change, the concentration ratio between the pairwise groups. ^c^ *p*, *T* test value, *p* < 0.05 represents statistically significant difference. ^d^ VIP, variable projection importance indicator. ^e^ “/” represents that it does not meet the *p* < 0.05, |r| > 0.50, VIP value is located in the top 10% of metabolites.

## Data Availability

The datasets generated during the current study are included in this published article, or they are available from the corresponding author on reasonable request.
